# Transmitral myectomy for mid-cavity obstruction and mitral replacement for rheumatic disease

**DOI:** 10.1016/j.xjtc.2025.01.023

**Published:** 2025-02-10

**Authors:** Fazal Khan, Mitra Khosravi-Flanigan, Bastiaan L. Kietselaer, Steve R. Ommen, Hartzell V. Schaff, Joseph A. Dearani, Paul C. Tang

**Affiliations:** a Department of Cardiovascular Surgery, Mayo Clinic, Rochester, Minn; b Department of Cardiovascular Medicine, Mayo Clinic, Rochester, Minn

**Figure:**
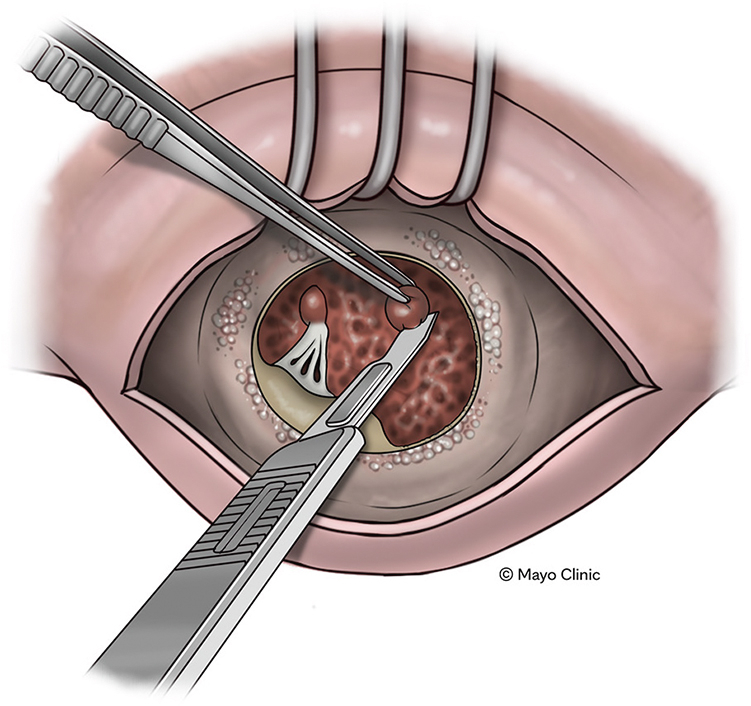
Papillary muscle resection to increase ventricular cavity volume.

**Central Message**

A transmitral approach is safe and effective for myectomy to relieve mid-cavitary obstruction with concomitant MV replacement. This avoids the risks of transaortic and transapical myectomies.

Left ventricular outflow tract (LVOT) obstruction in obstructive hypertrophic cardiomyopathy (HCM) can be related to the spectrum of anatomic components including the interventricular septum, subaortic curtain, fibrous trigones, subvalvular mitral apparatus, and systolic anterior motion of the mitral valve (MV).^1^ Approximately 10% of patients with obstructive HCM have mid-cavity obstruction from prominent mid-septal hypertrophy and papillary muscle abutment to the septum. During contraction, contact occurs between the interventricular septum with the left ventricular (LV) wall or prominent papillary muscle.^2^ Although transaortic septal myectomy is often performed to alleviate LVOT obstruction, this is limited by constrained visualization of the ventricular septum, particularly mid-cavity as well as access to the MV.^3^

Particularly in patients requiring mitral intervention, transmitral valve septal myectomy via the left atrium offers an attractive approach. This allows a panoramic view of the septum and allows for simultaneous assessment and intervention on MV pathologies.^3^ In situations where the native MV is to be preserved, the anterior leaflet is detached from trigone to trigone to allow access for myectomy and then reapproximated to the annulus with or without a patch afterward. However, if the MV is to be replaced and mid-cavitary obstruction needs to be addressed, then a transmitral approach is an excellent choice.

We discuss the advantages of the transmitral myectomy approach in the setting of MV replacement and mid-cavitary obstruction from HCM. Through a literature review, we highlight the efficacy and safety of transmitral myectomy for concomitant MV pathology and mid-cavity obstruction. The Mayo Clinic Institutional Review Board has deemed this report exempt from review.

## Case

**Figure 1:**
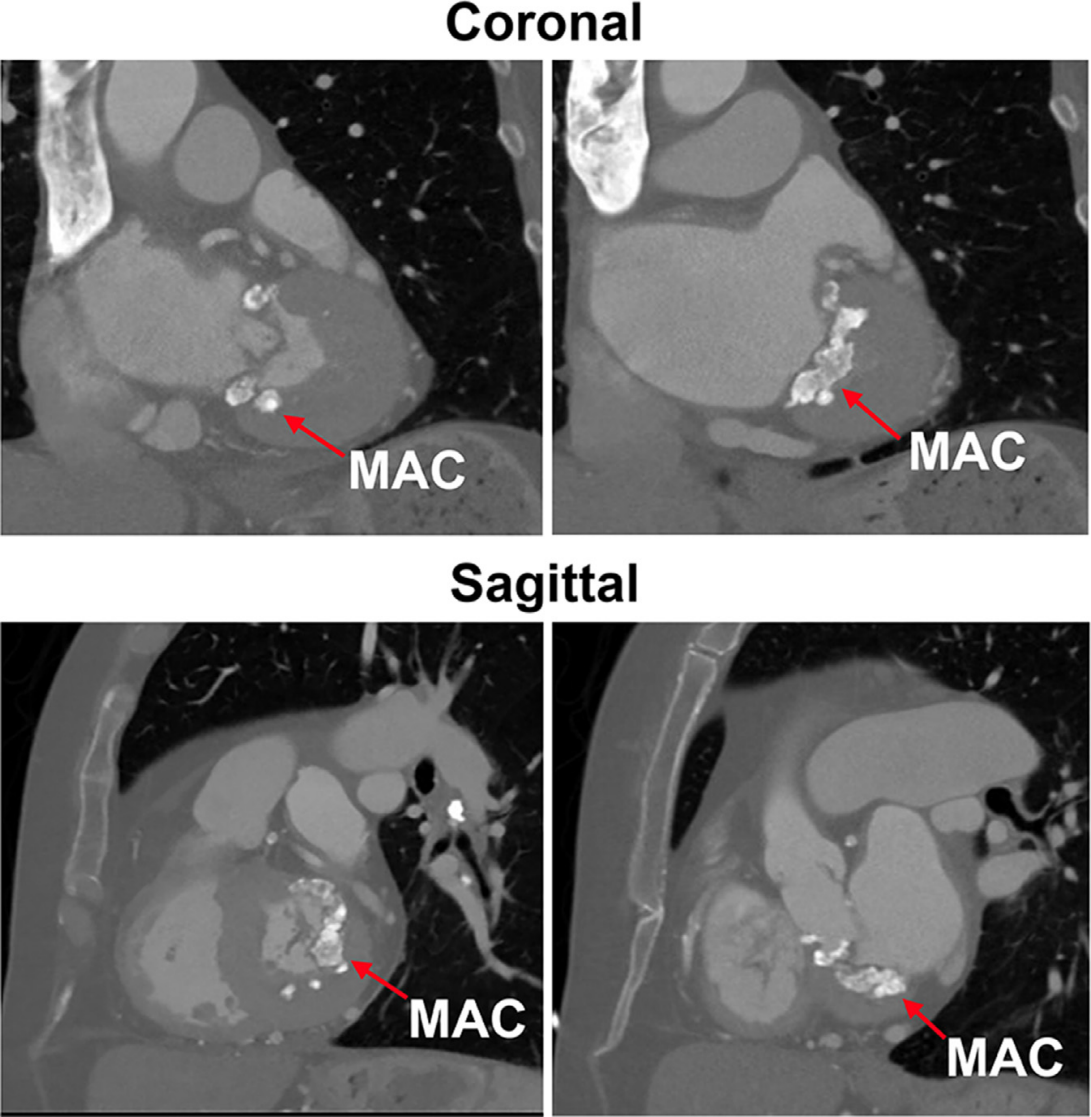
MAC shown on coronal and sagittal section on computed tomography. *MAC*, Mitral annular calcification.

A 76-year-old woman presents with progressive dyspnea and decreased exercise tolerance. She has a history of hypertension, primary biliary cirrhosis, hyperlipidemia, and hypothyroidism. The preoperative echocardiogram (Video 1) showed an LV ejection fraction of 70% with severe mitral stenosis (mean gradient 12 mm Hg, and area of 1.4 cm^2^) and moderate mitral regurgitation and associated severe circumferential mitral annular calcification (MAC). There was also severe mid-cavitary obstruction (mean gradient 60 mm Hg at rest) with septal thickness of 1.8 cm associated with severe concentric hypertrophy. The LVOT velocity was 1.6 m/s with a mean gradient of 12 mm Hg. Coronary angiography showed no significant coronary disease. Computed tomography confirmed heavy circumferential MAC (Figure 1). Her preference was for a mechanical prosthesis to minimize future interventions.

## Operative Technique

**Figure 2:**
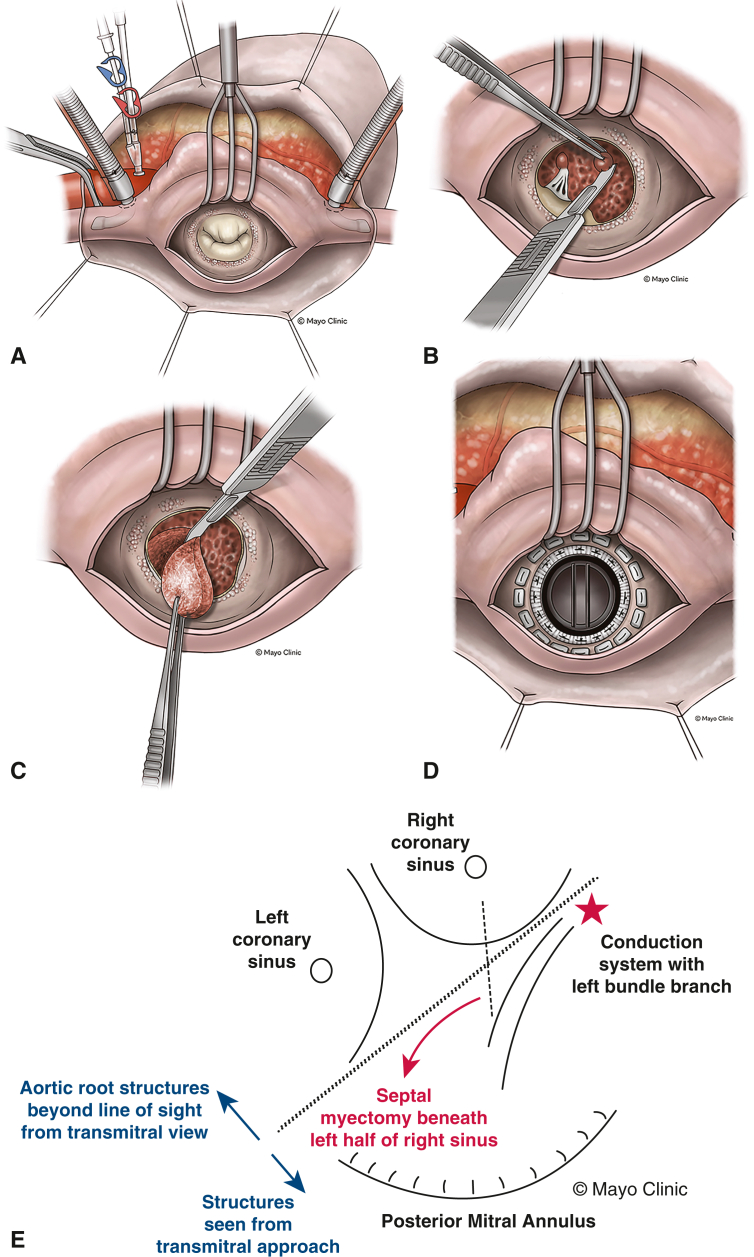
Mid-cavitary and septal myectomy with mitral replacement. A, Left atrial exposure of calcified MV and annulus. B, Resection of papillary muscle in mid-ventricular cavity. C, Septal myectomy from left atrial exposure where an incision was made approximately 5 mm below the center of the aorto-mitral curtain and extending leftward toward the posterolateral LV free wall. The myectomy depth was titrated based on knowing septal thickness and that the 10 blade used for resection is 8 mm in width. It is also important to resect the anterior mitral leaflet to avoid LVOT obstruction. D, Completed mechanical MV replacement. E, Pictorial of septal resection extent starting from beneath the left half of the right sinus away from the conduction system. This resection is then continued toward the left sinus demarcating the LV free wall.

Median sternotomy, central aortic access, biatrial cannulation, cardiopulmonary bypass, and cardioplegic arrest was performed. The MV was exposed through the left atrium via Sondergaard's groove (Figure 2, *A*). Dense MAC was encountered as anticipated. The thickened and calcified MV was excised leaving some of the posterior leaflet and subvalvar apparatus intact to support the atrioventricular groove. Other areas of the native annulus were carefully debrided with rongeurs to avoid fracturing the bulky calcium burden and reestablish a wide orifice. Inspection of the ventricular cavity revealed thick ventricular trabeculae and large papillary muscles with thick chordae. A 10 blade was used to excise thick trabeculae in the mid-cavity as well as portions of the bulky papillary muscle bodies. After debulking some of the papillary muscle and subvalvar apparatus, a papillary muscle with associated chords was left in place to maintain continuity with the posterior mitral annulus (Figure 2, *B*). A prominent bulge of septal muscle was also resected (Figure 2, *C*). The cavity appeared larger after the described myectomies. A 25-mm On-X mechanical valve was then implanted using 2-0 Ethibond transannular sutures placed while avoiding densely calcific areas (Figure 2, *D*).

The heart was weaned from cardiopulmonary bypass without difficulty. Postprocedural echocardiogram showed no mitral regurgitation with normal prosthetic MV function (mean gradient 3 mm Hg). There was no significant gradient in the LVOT by simultaneous needle measurements in the aorta and LV cavity. There was no significant residual mid cavitary gradient with excellent LV diastolic filling (Video 2). The patient was discharged home after an uncomplicated recovery.

## Discussion

A transmitral valve approach for mid-cavity septum hypertrophy in obstructive HCM patients dates back to the 1960s when Lillehei and Levy^4^ reported 2 successful cases. A transaortic approach often affords more limited visualization to the mid and distal LV cavity, so a transapical approach is often used for myectomies in these deeper regions.^2^ The transmitral approach involves incising the anterior leaflet for exposure and repairing the leaflet after myectomy.^5^ However, there is concern regarding long-term mitral competency due to disrupting the native MV followed by annular reattachment.

We report an excellent scenario for a transmitral approach to myectomy for mid-cavitary obstruction, which is the need for MV replacement and myectomy for the relief of mid-cavitary obstruction. Because the MV has to be replaced, there are no long-term concerns associated with leaflet incision and reattachment for mitral preservation. Furthermore, the subannular mitral apparatus can be aggressively resected to free up space for diastolic filling. This approach through the left atrium provides excellent visualization of the mid-ventricular cavity, thus allowing portions of the papillary apparatus and trabeculae to be readily resected. Thus, the transaortic or transapical approaches are not needed to reach the mid-ventricular cavity for this scenario in our experience. Furthermore, a septal myectomy can be effectively performed through the same transmitral exposure.

## Conclusions

We highlight a tailored surgical approach for addressing obstructive HCM in the setting of mitral pathologies requiring mitral replacement. Understanding the various surgical approaches to address obstructive HCM and concomitant mitral disease would be useful in the surgical armamentarium.

## Conflict of Interest Statement

P.C.T. is a noncompensated member of the Data Safety and Monitoring Board for the PRESERVE trial (XVIVO Inc.). All other authors reported no conflicts of interest.

The *Journal* policy requires editors and reviewers to disclose conflicts of interest and to decline handling or reviewing manuscripts for which they may have a conflict of interest. The editors and reviewers of this article have no conflicts of interest.

## Supplementary Data

Video 1 Preoperative transesophageal echocardiography. Video available at: https://www.jtcvs.org/article/S2666-2507(25)00062-8/fulltext.

Video 2 Postoperative transesophageal echocardiography. Video available at: https://www.jtcvs.org/article/S2666-2507(25)00062-8/fulltext.
