# Supplementary figures and images for: Transmitral myectomy for mid-cavity obstruction and mitral replacement for rheumatic disease
Source: JTCVS Tech. 2025 Feb 10;30:77–80. doi: 10.1016/j.xjtc.2025.01.023 (PMC11998312; doi:10.1016/j.xjtc.2025.01.023)

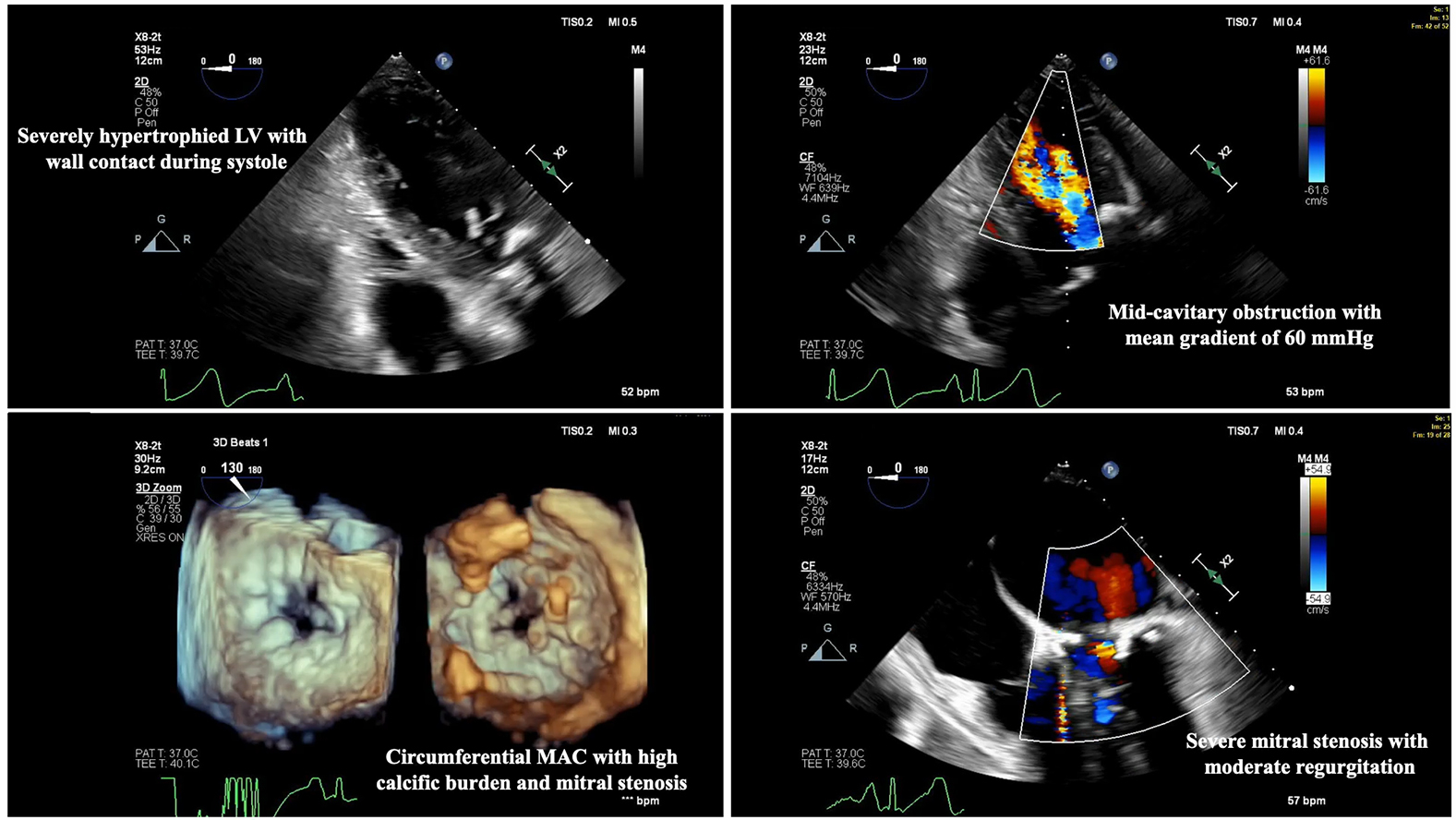

Supplement: Video 1 — Preoperative transesophageal echocardiography. Video available at: https://www.jtcvs.org/article/S2666-2507(25)00062-8/fulltext. [file fx2.jpg]

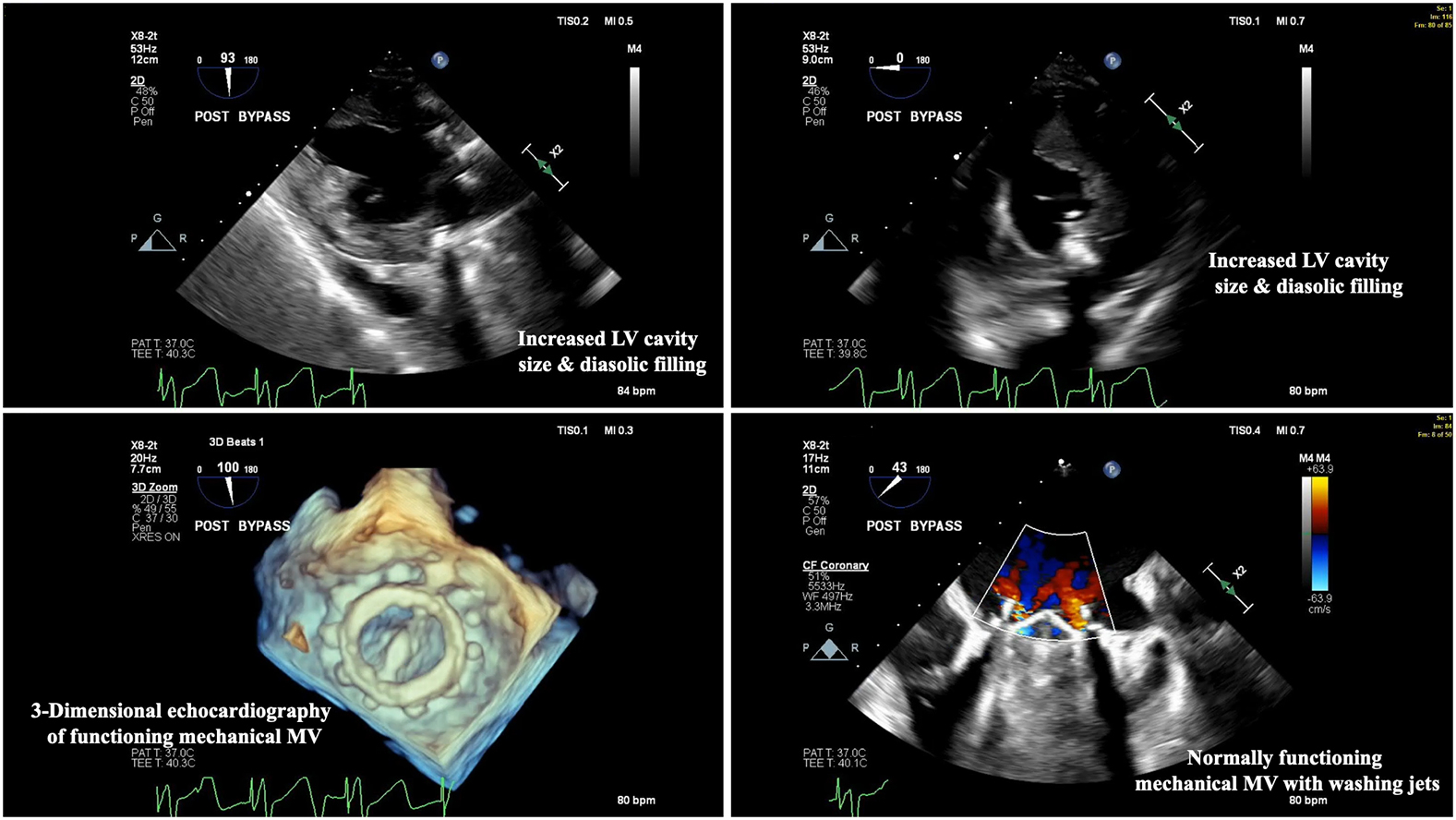

Supplement: Video 2 — Postoperative transesophageal echocardiography. Video available at: https://www.jtcvs.org/article/S2666-2507(25)00062-8/fulltext. [file fx3.jpg]
